# Primary Injuries and Secondary Organ Failures in Trauma Patients with Acute Kidney Injury Treated with Continuous Renal Replacement Therapy

**DOI:** 10.1155/2014/235215

**Published:** 2014-12-23

**Authors:** Sigrid Beitland, Ingrid Os, Kjetil Sunde

**Affiliations:** ^1^Department of Anaesthesiology, Division of Emergencies and Critical Care, Oslo University Hospital, Postboks 4956, Nydalen, 0424 Oslo, Norway; ^2^Institute of Clinical Medicine, Faculty of Medicine, University of Oslo, Postboks 1072, Blindern, 0316 Oslo, Norway; ^3^Division of Medicine, Department of Nephrology, Oslo University Hospital, Postboks 4956, Nydalen, 0424 Oslo, Norway

## Abstract

*Background*. Acute kidney injury (AKI) treated with continuous renal replacement therapy (CRRT) is a severe complication in trauma patients. The aim of the study was to assess primary traumatic injuries and secondary organ failures in severe posttraumatic AKI. *Methods*. Retrospective review of adult trauma patients admitted to the trauma centre at Oslo University Hospital Ullevål. Injury severity score (ISS) was used to assess the severity of primary injuries, and sequential organ failure assessment (SOFA) score was utilized to measure secondary organ failures. *Results*. Forty-two (8%) of 506 trauma patients admitted to intensive care unit developed AKI treated with CRRT, whereof 40 (95%) suffered blunt trauma mechanisms. Patients had extensive primary organ injuries with median (interquartile range) ISS 36 (27–49). The majority of the patients had respiratory (93% intubated) and cardiovascular (67% with inotropic and/or vasoactive medication) failure within 24 hours after admission. AKI was often part of multiple organ failure, most frequently respiratory and cardiovascular failure, affecting 33 (75%) and 30 (71%) of the patients, respectively. *Conclusion*. Trauma patients with AKI undergoing CRRT often had severe primary injuries due to blunt trauma. Most of them suffered from secondary multiple organ failure concomitant to AKI.

## 1. Introduction

Trauma is a leading cause of disability and death among young people in the developed world [1]. The primary injury often initiates tissue necrosis with release of intracellular muscular constituents, such as creatine kinase (CK) and myoglobin, into the systemic circulation [2]. Together with massive uncontrolled bleeding, coagulopathy, and infection, this may lead to tissue ischemia and ultimately contribute to life-threatening multiple organ failure (MOF) [3].

In general, acute kidney injury (AKI) is rare among trauma patients and is quite modestly described [3–5]. A multicenter study showed that trauma patients accounted for only 2% of severe AKI in the intensive care unit (ICU) [6]. Likewise, in a retrospective study of trauma patients admitted to our ICU at Oslo University Hospital Ullevål (OUHU), we found that only 8% developed AKI with need of CRRT [7]. The pathophysiology of posttraumatic AKI is quite complex, and only a few publications describe which types of traumatic injuries may eventually lead to AKI [8, 9]. Toxic substances from skeletal muscle necrosis lead to rhabdomyolysis, which, in combination with other prerenal, renal, and postrenal factors, is a major contributor to acute kidney injury (AKI) in trauma patients [10]. These toxic substances can partly be eliminated from the circulation by forced alkaline diuresis [2], but in the most severe cases, fluid overload and accumulation of metabolic waste products must be treated with renal replacement therapy (RRT), that is, dialysis [11]. In haemodynamic unstable ICU patients, continuous renal replacement therapy (CRRT) is often the preferred modality of RRT [12]. However, there is a debate upon the optimal timing of CRRT initiation in AKI. A systematic review and meta-analysis in general ICU patients concluded that early initiation of RRT may have a beneficial impact on survival [13], supported by some limited data in trauma patients [14]. Moreover, data from general ICU patients suggest that oliguric compared to nonoliguric severe AKI is associated with an unfavourable outcome [15]. Nevertheless, no previous study has reported the possible association between low diuresis and adverse outcome in trauma patients with severe AKI.

Thus, the aim of this retrospective study was to describe posttraumatic AKI treated with CRRT in adults focusing upon the primary traumatic injuries and secondary organ failures. We also assessed time from trauma to initiation of CRRT (early versus late CRRT) and diuresis at initiation of CRRT (oliguric versus nonoliguric AKI).

## 2. Material and Methods

### 2.1. Study Population and Definitions

OUHU is a major Scandinavian trauma centre currently admitting approximately 1800 trauma patients per year. It serves as a regional trauma centre for 2.7 million people, more than half the Norwegian population. Adult trauma patients (>18 years) admitted to OUHU between January 1, 1997, and December 31, 2006, were retrospectively reviewed, as a substudy of a previous report on the same patients [7]. Traumatized patients developing AKI and in the need of CRRT during their hospital stay were included in the study. Patients with chronic renal failure were excluded based on information about chronic renal failure or use of RRT in the medical history, or severely elevated creatinine and/or urea concentrations in blood sample analyses. Those receiving CRRT for other reasons than AKI were also excluded. The Norwegian Data Inspectorate (license number 06/01743-7) and the Regional Committee for Medical Research Ethics (approval number REK 1 408-06170 1.2006.2069) approved the study.

Simplified acute physiology score (SAPS) II was used to describe severity of illness [16], and injury severity score (ISS) was used to assess severity of injury [17]. Sequential organ failure assessment (SOFA) score was utilized to measure the organ failures at the initiation of CRRT [18]. Rhabdomyolysis was defined as a peak serum CK concentration above 10000 U/L. Oliguria was defined as urine output ≤500 mL per day, and early initiation of CRRT was defined as CRRT initiated ≤5 days after trauma. Multiple fractures were defined as three or more radiological confirmed fractures. We further registered intravenous infusions of dopamine, dobutamine, epinephrine, norepinephrine, and/or phenylephrine, and they were classified together as inotropic and/or vasoactive medications. One of the authors (SB) collected the data and assessed the extent of primary traumatic injuries.

### 2.2. Data Collection

Patients with a trauma responding for their present ICU stay were identified by searching all available diagnosis codes for trauma, excluding foreign bodies, late effects, and complications not directly related to the primary injury. AKI was developed and defined in the patients, using not only diagnosis codes for acute renal failure, but also procedure codes for CRRT. Different electronic databases were cross checked to ensure that no patients were missed, that is, the institutional hospital charts, local trauma registry, and ICU registry, and also the Norwegian national renal registry.

### 2.3. Statistical Analyses

Categorical data are presented as number (percent) and compared using a two-sided Pearson's chi-square test. Continuous data are presented as median (interquartile range (IQR)) and compared using a 2-tailed Mann-Whitney* U* test. The level ofstatistical significance was set at *P* < 0.05. Statistical analyses were performed in Statistical Package for Social Sciences (SPSS) for Windows, version 15.0 (SPSS Inc., Chicago, IL, USA).

## 3. Results

### 3.1. Patient Characteristics

Among 506 trauma patients admitted to the ICU, 42 (8%) developed AKI with need of CRRT and were included in the study. Median age was 46 years, and 86% were male. Seventeen (41%) patients had rhabdomyolysis, whereof 15 were treated with forced alkaline diuresis. None of the patients were dialysis-dependent three months after initiation of CRRT, and three-month mortality was 36% (Table 1).

### 3.2. Primary Traumatic Injuries

The patients were severely traumatized with median ISS 36 (IQR, 27–49). Blunt trauma was the injury mechanism in 40 (95%), mainly traffic accidents. Most of the patients had primary organ injuries in several body regions, most frequently in thorax and abdomen, affecting 30 (71%) and 27 (64%) of the patients, respectively. Orthopedic injuries were also common, with 22 (52%) of the patients having pelvis fractures and 32 (76%) multiple fractures. Primary injuries in the central nervous system affected nine (21%) of the patients (Table 1).

### 3.3. Primary Physiological Response and Performed Procedures

The trauma patients were severely ill with median SAPS II score 40 (IQR, 32–48). Within the first 24 hours after hospital admission, median maximum heart rate was 125 (IQR, 113–143) beats per minute, and median minimum systolic blood pressure was 68 (IQR, 55–72.5) mmHg. Inotropic and/or vasoactive medications were used in 28 (67%) of the patients, and the number of packed red blood cell (PRBC) transfusions was median 11 (IQR, 4–26). Furthermore, 39 (93%) of the patients were intubated. Even though they subsequently developed AKI with need of CRRT, the daily urine output at admission day was median 2420 (IQR, 1610–3580) mL. Within the first day, 38 (90%) and nine (21%) patients underwent surgical and radiological procedures, respectively (Table 2).

### 3.4. Secondary Organ Failures

**Figure 1:**
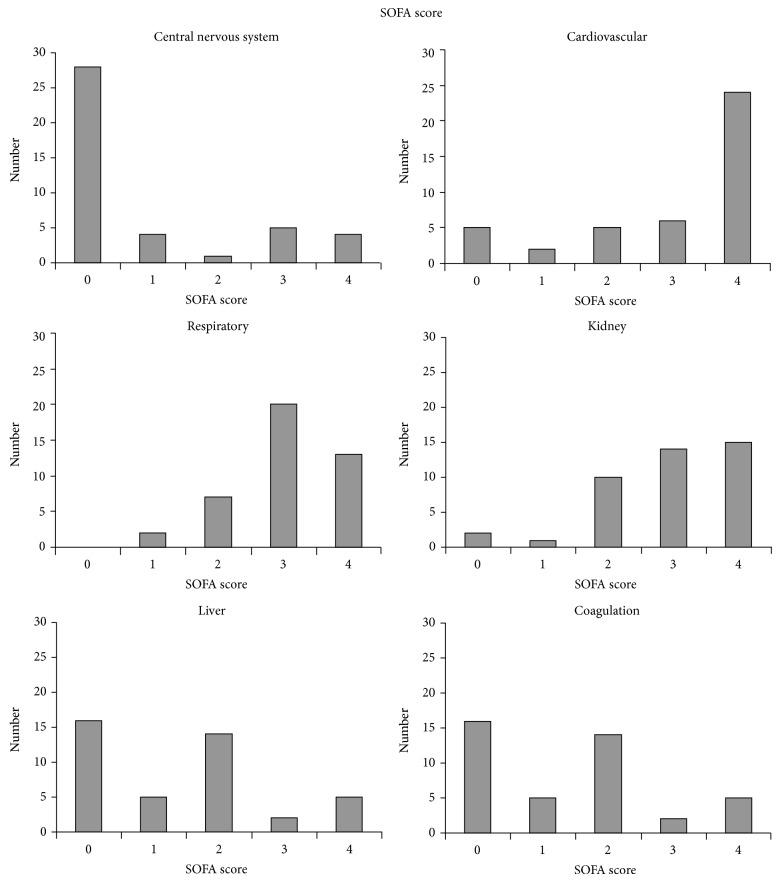
The organ failures measured by sequential organ failure assessment (SOFA) score in trauma patients with acute kidney injury and need of continuous renal replacement therapy (*n* = 42). Data is presented as number of patients having SOFA score 0–4 for the six measured organ functions at the time of initiation of continuous renal replacement therapy.

Time from trauma to initiation of CRRT among the 42 patients was median five (IQR, 3–11) days. The most frequent organ malfunctions were respiratory and cardiovascular failure, affecting 33 (75%) and 30 (71%) of the patients when assessed by SOFA score 3 or 4, respectively. Additionally, 29 (69%) patients had kidney failure when assessed by SOFA score 3 or 4, whereas failure of the central nervous system, liver, and/or coagulation system was rather infrequent (Figure 1).

### 3.5. Subgroup Analyses

Patients with early initiation of CRRT had significantly higher peak serum CK concentrations compared to the patients with late initiation of CRRT (median (IQR) 9643 (2775–43434) U/L versus 317 (101–2499) U/L, resp., *P* < 0.01). The number of PRBC transfusions, occurrence of MOF, and mortality were comparable (Table 3).

CRRT was initiated significantly earlier in patients with oliguric severe AKI than in patients with nonoliguric severe AKI (median (IQR) 4 (2–6.25) days versus 7 (3–13) days, resp., *P* < 0.01). Diuresis at admission day, occurrence of MOF, and mortality were similar (Table 3).

## 4. Discussion

We have shown in 42 trauma patients that AKI treated with CRRT was often a consequence of blunt trauma mechanisms with severe injuries to several body regions simultaneously. The majority of the patients had respiratory (93%) and cardiovascular (67%) failure within 24 hours after admission. Even though most patients seemed to have an adequate diuresis at admission day, they still developed AKI with need of RRT. There was a delay from the initial trauma to initiation of RRT of median five days. Severe AKI was often part of MOF, and the most frequent concomitant organ malfunctions were respiratory (75%) and cardiovascular (71%) failure, respectively.

AKI is a severe complication in trauma patients associated with a several-fold increase in hospital death; it is especially hazardous in the most severe cases where CRRT is being used [4, 19]. Even though only 0.1–8.4% of trauma patients with AKI are in the need of CRRT, the mortality in this subgroup is still 40–70% [8, 9, 14]. The high mortality rates in severe AKI have previously been studied in general ICU patients and can partly be explained by an early reduction in organ functions after RRT initiation [20]. Due to the complex pathophysiological mechanisms involved, posttraumatic AKI might still be different from other causes of AKI in the ICU. It is therefore important to achieve more information about this patient group to gain better understanding of the disease and ultimately apply this into change in clinical practice.

The present trauma patients with AKI undergoing CRRT were severely injured with a high ISS, and 64% had primary abdominal injuries. High ISS scores have previously been found in patients with posttraumatic AKI [4, 10, 19] and identified as an independent risk factor for AKI [10, 19]. However, the exact trauma mechanisms and primary organ injuries are, in previous studies, modestly described, except for one study showing a dominating blunt trauma mechanism [8]. It is therefore still unsettled to which extent the direct injuries of the kidneys and urinary tract might contribute to AKI, and also the effects of postrenal components such as retroperitoneal hematomas and/or extraperitoneal pelvic packing [7].

Severity of illness [4, 19] and extensive bleeding [4] have previously been associated with increased risk of posttraumatic AKI. The majority of the present patients needed early surgical, radiological, and other procedures. Moreover, they had severe physiological derangement with high rates of respiratory and cardiovascular failure, factors associated with increased mortality in posttraumatic severe AKI [4, 21]. As previously described, posttraumatic AKI might be an early biological marker of severity of disease and subsequent MOF [5].

Although it seems obvious to detect AKI as early as possible, we found no significant association between the time of initiation of CRRT and mortality. However, the number of patients was low. Previous data do indicate that early initiation of RRT may have a beneficial impact on survival in general ICU patients [13], and this was also true in a 15-year-old study in 100 trauma patients [14]. Indeed, prospective clinical studies comparing early versus later CRRT initiation in trauma patients are warranted. An interesting finding in the present study was the significantly higher peak serum CK concentrations in patients with early initiation of CRRT compared to the patients with late initiation of CRRT. We believe that this was due to rhabdomyolysis with massive tissue necrosis and early need of CRRT due to hyperkalemia and fluid overload.

In a study of general ICU patients, oliguric severe AKI was associated with worse renal outcomes and mortality compared to nonoliguric severe AKI [15]. These findings could not be confirmed in the present study, which again can be contributed to the low numbers of patients. However, we found that patients with oliguria required RRT earlier than those nonoliguric, and this has also previously been confirmed in a study of severe AKI in general ICU patients [15]. Further, we believe that the relatively high diuresis measured on admission day in our study population was in part due to treatment with forced alkaline diuresis, with both fluids and diuretics.

This study has some important limitations as it presents only a small number of patients from a single trauma centre. Additionally, only the most severe type of posttraumatic AKI is included, that is, those receiving CRRT. Combined with a wide variation in the practical performance of CRRT among institutions, the external validity of the findings probably is limited. Further, the subgroup analyses should be interpreted with caution because of the small number of patients in each group with limited statistical power (type II error). The definitions of the subgroups, that is, oliguric/nonoliguric and early/late, may also vary among studies. Indeed, prospective studies in this complex subgroup of ICU patients are needed in the future for better-targeted treatment and reduction in mortality.

## 5. Conclusions

Trauma patients with AKI treated with CRRT were often victims of blunt trauma with severe primary organ injuries in several body regions. There was a time delay of several days from the initial trauma to the need of CRRT in most patients, and AKI was frequently associated with MOF.

## Figures and Tables

**Table 1:** Patient characteristics, trauma mechanism, and organ injuries in trauma patients with acute kidney injury treated with continuous renal replacement therapy (*n* = 42).

Demographic data	
Age (years)	46 (29–64)
Male gender	36 (86)
SAPS II score	40 (32–48)
ISS score	36 (27–49)
SOFA score	13 (12–15)
Time from trauma to CRRT (days)	5 (3–11)
Duration of any RRT (days)	6 (4–15)

Trauma mechanisms	
Blunt trauma	40 (95)
Car	17 (41)
Squeeze	10 (24)
Motorcycle	7 (17)
Pedestrian	2 (5)
Fall	2 (5)
Gun	2 (5)
Train	1 (2)
Explosion	1 (2)

Primary organ injuries	
Central nervous system	9 (21)
Thorax	30 (71)
Abdomen	27 (64)
Pelvis	22 (52)
Multiple fractures	32 (76)

Outcome	
Intensive care unit length of stay (days)	26 (12–46)
Dialysis-dependent 3 months	0 (0)
3-month mortality	15 (36)

Categorical data are presented as number (percent) and continuous data as median (interquartile range). SAPS II: simplified acute physiology score II.

ISS: injury severity score. SOFA: sequential organ failure assessment.

CRRT: continuous renal replacement therapy. RRT: renal replacement therapy. RIFLE: risk, injury, failure, loss, and end-stage renal disease.

**Table 2:** Organ functions and performed procedures in trauma patients with acute kidney injury treated with continuous renal replacement therapy (*n* = 42).

Organ function at admission day	
Maximum heart rate	125 (113–143)
Arrhythmias	3 (7)
Minimum systolic blood pressure	68 (55–72.5)
Inotropic and/or vasoactive medications	28 (67)
Intubation	39 (93)
Highest FiO_2_	0.7 (0.5–0.9)
Highest peak inspiratory pressure (cmH_2_O)	29 (26–32)
Highest positive end expiratory pressure (cmH_2_O)	8 (6–10)
Diuresis (mL)	2420 (1610–3580)
Lowest blood base excess (mmol/L)	−10 (−5–−10)
Highest blood glucose (mmol/L)	10 (8–12)

Surgical, radiological, or other procedures at admission day	
Fasciotomy	11 (26)
Amputation	10 (24)
Endovascular embolization	9 (21)
Vascular surgery	6 (14)
Thoracotomy	6 (14)
Laparotomy	16 (38)
Peritoneal lavage	7 (17)
Peritoneal lavage and laparotomy	3 (7)
Orthopedic surgery	18 (43)
Total operative time (minutes)	265 (132–401)
PRBC transfusions (number)	11 (4–26)

Categorical data are presented as number (percent) and continuous data as median (interquartile range). FiO_2_: fraction of inspired oxygen.

PRBC: packed red blood cell.

**Table 3:** Subgroups analyses of trauma patients with acute kidney injury treated with continuous renal replacement therapy (*n* = 42).

Early versus late initiation of CRRT				
Days from trauma to initiation of CRRT ≤5 days (early) or >5 days (late)				

	Overall (*n* = 42)	Early (*n* = 23)	Late (*n* = 19)	*P*=

PRBC transfusions (number)	11 (4–26)	10 (3–23)	13.5 (5.5–26.25)	0.45
Serum creatine kinase (U/L)	3814 (575–25487)	9643 (2775–43434)	317 (101–2499)	<0.01
Multiple organ failure	27 (64)	16 (69)	11 (58)	0.43
Mortality	15 (36)	6 (26)	9 (47)	0.15

Oliguric versus nonoliguric acute kidney injury				
Daily diuresis at initiation of CRRT ≤500 mL (oliguric) or >500 mL (nonoliguric)				

	Overall (*n* = 42)	Oliguric (*n* = 16)	Nonoliguric (*n* = 26)	*P*=

Diuresis admission day (mL)	2420 (1610–3580)	2020 (1375–2808)	2666 (1858–3680)	0.81
Days to CRRT^*^	5 (3–11)	4 (2–6.25)	7 (3–13)	<0.01
Multiple organ failure	27 (64)	13 (81)	14 (58)	0.07
Mortality	15 (36)	7 (44)	8 (31)	0.39

Categorical data are presented as number (percent) and compared using 2-sided Pearson's Chi-squared test. Continuous data are presented as median (interquartile range) and compared using 2-tailed Mann-Whitney *U* test. CRRT: continuous renal replacement therapy. PRBC: packed red blood cell.

^*^Days from traumatic insult to initiation of CRRT.

## References

[1] Polinder S., Haagsma J. A., Toet H., Brugmans M. J. P., van Beeck E. F. (2010). Burden of injury in childhood and adolescence in 8 European countries. *BMC Public Health*.

[2] Holt S. G., Moore K. P. (2001). Pathogenesis and treatment of renal dysfunction in rhabdomyolysis. *Intensive Care Medicine*.

[3] Lehmann U., Grotz M., Regel G., Rudolph S., Tscherne H. (1995). Does the initial treatment of multiple trauma patients influence the development of multiple organ failure? Evaluation of the preclinical and clinical data of 1112 patients with multiple injuries. *Unfallchirurg*.

[4] Bihorac A., Delano M. J., Schold J. D., Lopez M. C., Nathens A. B., Maier R. V., Layon A. J., Baker H. V., Moldawer L. L. (2010). Incidence, clinical predictors, genomics, and outcome of acute kidney injury among trauma patients. *Annals of Surgery*.

[5] Wohlauer M. V., Sauaia A., Moore E. E., Burlew C. C., Banerjee A., Johnson J. (2012). Acute kidney injury and posttrauma multiple organ failure: the canary in the coal mine. *Journal of Trauma and Acute Care Surgery*.

[6] Uchino S., Kellum J. A., Bellomo R., Doig G. S., Morimatsu H., Morgera S., Schetz M., Tan I., Bouman C., Macedo E., Gibney N., Tolwani A., Ronco C. (2005). Acute renal failure in critically ill patients: a multinational, multicenter study. *The Journal of the American Medical Association*.

[7] Beitland S., Moen H., Os I. (2010). Acute kidney injury with renal replacement therapy in trauma patients. *Acta Anaesthesiologica Scandinavica*.

[8] Morris J. A., Mucha P., Ross S. E. (1991). Acute posttraumatic renal failure: a multicenter perspective. *Journal of Trauma*.

[9] Regel G., Lobenhoffer P., Grotz M., Pape H. C., Lehmann U., Tscherne H. (1995). Treatment results of patients with multiple trauma: an analysis of 3406 cases treated between 1972 and 1991 at a German Level I Trauma Center. *Journal of Trauma*.

[10] Vivino G., Antonelli M., Moro M. L., Cottini F., Conti G., Bufi M., Cannata F., Gasparetto A. (1998). Risk factors for acute renal failure in trauma patients. *Intensive Care Medicine*.

[11] Pannu N., Klarenbach S., Wiebe N., Manns B., Tonelli M. (2008). Renal replacement therapy in patients with acute renal failure: a systematic review. *JAMA—Journal of the American Medical Association*.

[12] McCunn M., Reynolds H. N., Reuter J., McQuillan K., McCourt T., Stein D. (2006). Continuous renal replacement therapy in patients following traumatic injury. *International Journal of Artificial Organs*.

[13] Karvellas C. J., Farhat M. R., Sajjad I. (2011). A comparison of early versus late initiation of renal replacement therapy in critically ill patients with acute kidney injury: a systematic review and meta-analysis. *Critical Care*.

[14] Gettings L. G., Reynolds H. N., Scalea T. (1999). Outcome in post-traumatic acute renal failure when continuous renal replacement therapy is applied early vs. late. *Intensive Care Medicine*.

[15] Morgan D. J. R., Ho K. M. (2010). A comparison of nonoliguric and oliguric severe acute kidney injury according to the risk injury failure loss end-stage (RIFLE) criteria. *Nephron—Clinical Practice*.

[16] Le Gall J.-R., Lemeshow S., Saulnier F. (1993). A new simplified Acute Physiology Score (SAPS II) based on a European/North American multicenter study. *Journal of the American Medical Association*.

[17] Baker S. P., O'Neill B., Haddon W., Long W. B. (1974). The injury severity score: a method for describing patients with multiple injuries and evaluating emergency care. *The Journal of Trauma*.

[18] Vincent J. L., de Mendonça A., Cantraine F., Moreno R., Takala J., Suter P. M., Sprung C. L., Colardyn F., Blecher S. (1998). Use of the SOFA score to assess the incidence of organ dysfunction/failure in intensive care units: results of a multicenter, prospective study. *Critical Care Medicine*.

[19] Bagshaw S. M., George C., Gibney R. T. N., Bellomo R. (2008). A multi-center evaluation of early acute kidney injury in critically ill trauma patients. *Renal Failure*.

[20] Cappi S. B., Sakr Y., Vincent J. L. (2006). Daily evaluation of organ function during renal replacement therapy in intensive care unit patients with acute renal failure. *Journal of Critical Care*.

[21] Tran D. D., Cuesta M. A., Oe P. L. (1994). Acute renal failure in patients with severe civilian trauma. *Nephrology Dialysis Transplantation*.

